# Epidemiology and clinical profile of individuals with cleft lip and palate utilising specialised academic treatment centres in South Africa

**DOI:** 10.1371/journal.pone.0215931

**Published:** 2019-05-09

**Authors:** Phumzile Hlongwa, Jonathan Levin, Laetitia C. Rispel

**Affiliations:** 1 School of Oral Health Sciences, Faculty of Health Sciences, University of the Witwatersrand, Johannesburg; 2 School of Public Health, Faculty of Health Sciences, University of the Witwatersrand, Johannesburg; 3 Centre for Health Policy & DST/NRF SARChI Chair, School of Public Health, Faculty of Health Sciences, University of the Witwatersrand, Johannesburg; Agha Khan University, UNITED REPUBLIC OF TANZANIA

## Abstract

**Objective:**

The study was conducted to determine the epidemiology and clinical profile of individuals with cleft lip and/or palate (CLP) utilizing specialized academic treatment centres in South Africa’s public health sector.

**Materials and methods:**

The Human Research Ethics Committee of the University of the Witwatersrand in Johannesburg provided ethical approval for the study. We conducted a retrospective record review of all cases of CLP treated at the specialised academic centres for the two-year period from 1 January 2013 until 31 December 2014. We used a structured, pre-tested record review form to obtain demographic, clinical and treatment information on each CLP case. We used Stata 13 to analyse the data and conducted statistical tests at 5% significance level.

**Results:**

We analysed 699 records of individuals with CLP. The estimated prevalence of CLP in the South African public health sector was 0.3 per 1000 live births, with provincial variation of 0.1/1000 to 1.2/1000. The distribution of clefts was: 35.3% cleft palate; 34.6% cleft lip and palate; 19.0% cleft lip and other cleft anomalies at 2%. Of the total number of CLP, 47.5% were male and 52.5% female, and this difference was statistically significant (p<0.001). The majority of clefts occurred on the left for males (35.5%) and palate for females (43.4%), with a male predominance of unilateral cleft lip and palate (53.3%).

**Conclusion:**

The study findings should inform the implementation of South Africa’s planned birth defect surveillance system and health service planning for individuals with CLP.

## Introduction

Congenital anomalies, defined as abnormalities of structure, function, or metabolism that are present at birth, are a major public health concern due to their life threatening nature or the potential to result in disability or death. Worldwide, it is estimated that 303 000 new-born infants die within four weeks of birth every year, due to congenital anomalies [1]. Clefting of the lip with or without palate (CLP) is the most common congenital craniofacial anomaly with the global prevalence estimated at 1 in 700 live births [2]. Orofacial clefts can occur on the lip only (CL), alveolar (CA), involve both lip and alveolar, affect the palate (CP) or involve both lip and palate (CLP). A cleft of the lip and/ or palate is serious, as it also affects negatively an individual’s self-esteem, social skills, and behaviour [3–5].

The prevalence of CLP differs according to gender, ethnicity, and socio-economic status [6]. Boys are more affected than girls with a reported ratio of 2:1 with cleft lip and/or cleft lip and palate, whilst females have a slightly greater risk for cleft palate only [7].

In many high-income countries (HICs), active surveillance systems are in place, and several CLP studies have been conducted that provide epidemiological trends and prevalence estimates [6, 8–11]. Several studies have reported on access and utilization of treatment and health care services for CLP [12–16]; standards and quality of care and long term health outcomes [17]; the clinical profile of cases, and the composition and interaction among healthcare team members in the treatment of CLP [18–21].

There is an emerging body of literature on CLP in low-and-middle-income countries (LMICs), focusing on the epidemiology of CLP [22–29], treatment and care of individuals with CLP, health care access, service challenges, and resource constraints [30–33]. The City of Bauru in Brazil has developed a centre of excellence for the comprehensive management of individuals with CLP more than 40 years ago [34]. A review of challenges in CLP care in Africa [35] underscored the lack of reliable data on the prevalence of CLP because most of the reported studies are hospital-based [27, 36, 37].

In many African countries, active population based surveillance systems are not available. Prevalence is estimated from hospital-based data, and ranges from 0.2/1000 live births in Ethiopia [38], 0.5/1000 in Nigeria [39], 0.8/1000 in Uganda [22] and 1.7/1000 reported in Kenya [40]. A community household survey in South East Ghana found an estimated prevalence of 6.3/1000 people with CLP [41], however CLP was measured through community self-reporting rather than clinical examination. Community reporting of orofacial cleft is influenced by context and the community’s description of the cleft which may be contrary to the scientific description of orofacial cleft phenotypes [42]. A recent study conducted in Democratic Republic of Congo reported an incidence of 0.8 per 1000 live births for non-syndromic CLP [43].

In South Africa, earlier studies on prevalence of CLP were conducted in Cape Town [23, 24], Johannesburg [30] and Pretoria [25, 26] in the late 1980s. The reported prevalence ranged from 0.1 to 0.4 per 1000 live births. However, these studies were conducted more than 30 years ago and they predate democracy in 1994. Furthermore, the studies focused on three major South Africa’s cities, and not all the specialised academic treatment centres were included. Importantly, a study that examined the causes of under-five mortality rates found that the proportion of deaths due to non-natural causes, congenital disorders and non-communicable diseases has increased [44]. In light of the dearth of scholarly studies on the epidemiology of CLP in South Africa, we conducted this study to determine the epidemiology and clinical profile of individuals with cleft lip and palate utilising specialised academic treatment centres in South Africa. It is part of a doctoral study on the epidemiology and care of individuals with CLP in South Africa.

## Methods

### Ethical considerations

The Human Research Ethics Committee (Medical) of the University of the Witwatersrand in Johannesburg provided ethical approval for the study to review patient medical records. All personal identifiers were removed from the records, hence no informed patient consent was required. Each specialised academic treatment centre also provided approval to access and review the patient medical records.

The principal investigator (PH) is registered with the Health Professional Council of South Africa as an orthodontist, and is familiar with all the principles of patient confidentiality in medical records. Only the PH had access to the relevant records for the study period. The medical records at each centre were assessed in a private area and never left unattended. The principal investigator allocated each patient record a unique identifier and no patient name or any other form of identification was recorded on the data collection form. The data containing unique numbers were kept on a password-protected computer.

### Study sites and setting

South Africa’s public health sector provides health care to an estimated 83% of the population, while the private health sector provides care to a minority (17%) of the population with private health insurance [45]. Public sector hospitals are categorised into five types, namely:—district hospitals; regional hospitals; tertiary hospitals; central hospitals and specialised hospitals [46]. There are ten central hospitals situated in six of South Africa’s nine provinces. These central hospitals are attached to Health Science Faculties, and serve as teaching centres for the training of health professionals. These central hospitals also provide tertiary hospital services and serve as referral facilities for primary and secondary health facilities, and in some cases as specialised centres for referral of complicated medical conditions from neighbouring provinces [46].

**Fig 1:**
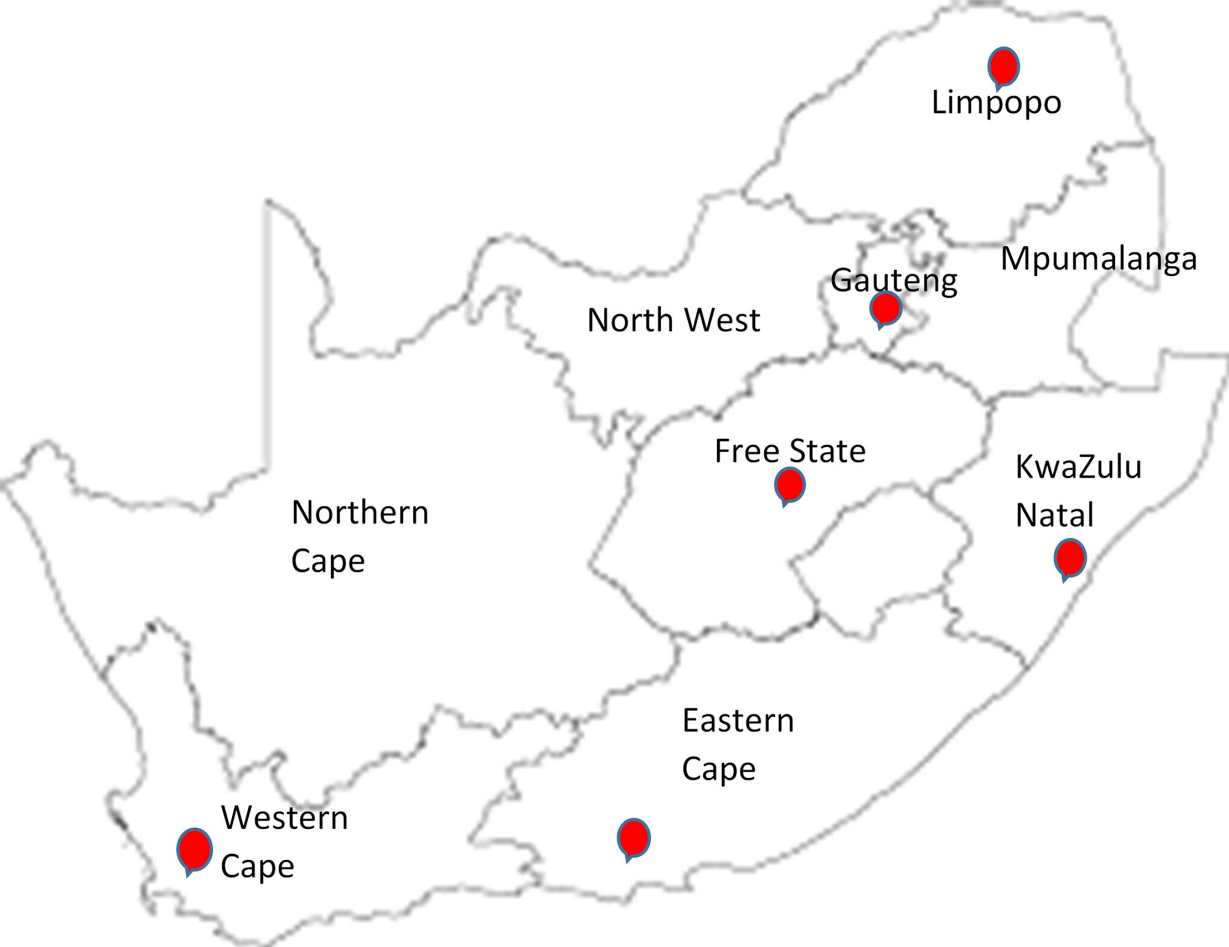
Map of South Africa showing provinces for study settings [47].

The study setting consisted of 11 specialised academic centres (nine central hospitals and two specialised dental hospitals) with multi-disciplinary teams of health professionals who provide care to individuals with CLP. These 11 centres are situated in six of South Africa’s nine provinces as shown in Fig 1, which included three mixed urban-rural provinces. We selected these centres because they cover all those individuals who obtain care for CLP in South Africa’s public health sector (83% of the population), and to generate new knowledge that will contribute to improvements in health care in the public health sector of South Africa.

In Gauteng Province, five CLP care centres were included in the study: Charlotte Maxeke Johannesburg Academic Hospital, Chris Hani Baragwanath Hospital, Dr George Mukhari Hospital, Pretoria Dental Hospital and Steve Biko Academic Hospital. In the Western Cape Province, the Red Cross War Memorial Children’s and Tygerberg Hospitals were included. One centre in each of the following provinces was included in the study: KwaZulu Natal Province—Inkosi Albert Luthuli Central Hospital; Eastern Cape Province—Nelson Mandela Academic Hospital; Free State Province—Universitas Hospital and Limpopo Province–Polokwane/Mankweng Hospital Complex.

### Study population and sampling

All clinical records for the selected study period, 1 January 2013 to 31 December 2014, constituted the study sample. At each of the CLP specialised care centres, we selected the clinical records of all cleft individuals that visited these academic centres during the study period. Because individuals with CLP make numerous visits to these centres over a prolonged period, care was taken to record each individual only once, in order to avoid duplications.

A structured, pre-tested record review form (S1 File), was used to obtain demographic, clinical and treatment information on each CLP individual. We recorded the “race” of individuals with CLP from a list of pre-defined categories: Black African, Coloured, Indian or Asian, White and other. Although we do not want to give credibility to the apartheid roots of these group classifications, years of systematic, legislated racism continue to shape socio-economic circumstances and access to care in post-apartheid South Africa [48]. The principal investigator (PH) extracted the information from the CLP medical records.

Each academic centre was allocated a unique identifier to ensure anonymity. The cleft types were classified into the various broad categories: Bilateral cleft lip and palate (BCLP), left cleft lip and palate (LCLP), right cleft lip and palate (RCLP), bilateral cleft lip only (BCL), left cleft lip only (LCL), right cleft lip only (RCL), cleft palate only (CP), cleft of alveolar only (CA), cleft lip and alveolar (CLA).

### Data management and analysis

The data was imported into STATA 13 for descriptive and inferential statistical analyses. All statistical tests were conducted at 5% significance level.

The total number of live births for the two year period (January 2013-December 2014) was obtained from Statistics South Africa [49].

We estimated three different CLP prevalence rates: overall prevalence rate; prevalence rate adjusted for the private health sector utilisation and missing data in one specialised centre; and provincial prevalence rates.

Overall prevalence rate: The total number of individuals with orofacial clefts at the specialised academic care centres, (numerator data) was divided by the total number of live births during the study period (denominator data) and the value multiplied by 1000.

Prevalence rate adjusted for the private health sector utilisation and missing data in one centre: In order to adjust for the 17% of the South African population covered by private health insurance [45], we assumed that 17% of all live births occur in the private health sector. We further calculated that 2% of births occurred in the specialised centre with missing data. During the study period, 43 240 live births [50] were recorded at that centre. The denominator was adjusted by subtracting 19% (private sector and missing data from one centre) from the total number of live births. Therefore, the prevalence of CLP was estimated by dividing the numerator with the adjusted denominator and multiplied by 1000.

Provincial prevalence estimates: We also calculated the prevalence of CLP in each province. The denominator (number of live births) was adjusted for each province to take account of private sector utilization. The proportion of provincial population with private health insurance ranged from 9% to 28% [45]. In the case of Gauteng Province, the denominator was also adjusted to take account of the centre with missing data. Following adjustments, the prevalence was calculated by dividing the number of CLP individuals in each province (numerator) by the number of live births (adjusted denominator) in that province during the study period, multiplied by 1000.

## Results

We reviewed 717 CLP records from 10 specialised academic centres, 18 were excluded from the study because of incomplete data, and the final sample was 699 records.

The majority of CLP cases (45.6%) were treated in four centres located in Gauteng Province.

### Profile of individuals utilising specialised academic centres

Table 1 shows the demographic characteristics of the individuals utilising the specialised academic centres. Cleft distribution by population groups showed majority for Black African, followed by White, Coloured and Indians respectively. More females presented with CLP compared to males. The majority of individuals treated in these centres (97%) were South Africans with a small percentage from neighbouring countries.

**Table 1:** Demographic characteristics of the CLP individuals.

Characteristics	Sample size
CLP median age at consultation in months (IQR)	3 (0.75–13)
**Gender**	**n = 694**
Male	330 (47.5%)
Female	364 (52.5%)
**Race**	**n = 687**
Black African	448 (65.1%)
Coloured	94 (13.6%)
Indian	37 (5.4%)
White	109 (15.9%)
**Nationality**	**n = 690**
South African	669 (97%)
Non- South African	21 (3%)
**CLP by Province of birth**	**n = 694**
Eastern Cape Province	18 (2.6%)
Free State Province	108 (15.6%)
Gauteng Province	202 (29.1%)
KwaZulu Natal Province	66 (9.5%)
Limpopo Province	60 (8.7%)
Mpumalanga Province	52 (7.5%)
North West Province	22 (3.1%)
Northern Cape Province	2 (0.3%)
Western Cape Province	146 (21%)
Non- South Africans	18 (2.6%)
**Number of CLP per Academic Centre**	**n = 699**
SITE 2	93 (13.3%)
SITE 3	33 (4.7%)
SITE 4	52 (7.4%)
SITE 5	141 (20.2%)
SITE 6	79 (11.3%)
SITE 7	70 (10%)
SITE 8	62 (8.9%)
SITE 9	16 (2.3%)
SITE 10	122 (17.5%)
SITE 11	31 (4.4%)

Accounting for missing data:—From 699 records, only 694 records had gender and province of birth indicated in them, 690 records had nationality shown, and race was recorded in 687 records. One centre, SITE 1, was excluded because there were no records available.

### Estimated CLP prevalence

The estimated overall prevalence rate of CLP was 0.3 per 1000 live birth calculated from 2,300 897 live births during the study period [49]. Prevalence rate adjusted for the private health sector utilisation and missing data in one centre was 0.4 per 1000 live births.

The prevalence of CLP per province shown in Table 2, ranged from 0.1 to 1.2 per 1000 live births. The highest prevalence was in the Free State Province and the lowest in the Eastern and Northern Cape Provinces. Although the majority of CLP were recorded in Gauteng Province, the estimated prevalence rate in this province was 0.5 per 1000 live births.

**Table 2:** Prevalence of CLP in each province.

Provinces	Number of CLP	Proportion on private health care*	Adjusted denominator**	Prevalence per 1000 live births
Eastern Cape Province	18	11%	235 247	0.1
Free State Province	108	18%	93 134	1.2
Gauteng Province***	202	28%	384 406	0.5
KwaZulu Natal Province	66	13%	412 034	0.2
Limpopo Province	60	9%	245 075	0.2
Mpumalanga Province	52	15%	150 813	0.3
North West Province	22	15%	130 298	0.2
Northern Cape Province	2	20%	43 374	0.1
Western Cape Province	146	26%	158 081	1.0

*Proportion on private health care in each Province [49]

** Adjusted denominator = number of live births adjusted by subtracting percentage to account for private sector utilisation [45]

*** For Gauteng Province, the denominator was also adjusted by 7% to account for missing data in one centre [50].

Table 3 shows the profile of clefting at academic treatment centres. Cleft palate only (CP) was the most predominant type of cleft followed by cleft lip and palate (CLP) and the cleft lip (CL). Other cleft abnormalities included midline facial cleft (2), lateral facial cleft (6), and syndromes (6).

**Table 3:** Profile of clefting at academic treatment centres.

Description	Frequency (Percentage)
**Cleft type**	**n = 699**
Cleft lip	133 (19.03%)
Cleft palate	247 (35.34%)
Cleft lip and palate	305 (43.63%)
Other	14 (2%)
**Cleft description**	**n = 699**
Unilateral	322 (46.07%)
Bilateral	114 (16.31%)
Palate	247 (35.34%)
Midline	2 (0.29%)
Other	14 (2%)
**Cleft laterality**	**n = 699**
Left	228 (32.62%)
Right	94 (13.45%)
Bilateral	114 (16.31%)
Palate	247 (35.34%)
Midline	2 (0.29%)
Other	14 (2%)
**Cleft position**	**n = 699**
Lip	99 (14.16%)
Alveolar	3 (0.43%)
Palate	247 (35.34%)
Cleft lip and palate	305 (43.63%)
Lip and alveolar	31 (4.43%)
Other	14 (2%)

Cleft distribution by gender and types is shown in Table 4. From 694 clefts, there were more CP in females than males whilst CLP was predominant in males. Unilateral clefts occurred most frequently in males compared to females. The left side dominated the occurrence of clefts compared to the right side in both CL and unilateral CLP for both genders. Statistically significant differences (*p*<0.001) were observed for cleft type, distribution and location between males and females.

**Table 4:** Distribution of types of clefts by gender.

n = 694	Male n (Col %)	Female n (Col %)
**Cleft type**		
Cleft lip	65 (19.7%)	66 (18.1%)
Cleft palate	88 (26.7%)	158(43.4%)
Cleft lip and palate	174 (52.7%)	129 (35.4%)
Other	3 (0.9%	11 (3%)
Total	330 (100%)	364 (100%)
**Cleft description**		
Unilateral	176 (53.3%)	142 (39%)
Bilateral	63 (19.1%)	53 (14.6%)
Palate	88 (26.7%	158 (43.4%)
Other	3 (0.9%)	11 (3%)
Total	330 (100%)	364 (100%)
**Cleft laterality**		
Left	117 (35.5%)	108 (29.7%)
Right	59 (18.5%)	34 (9.3%)
Left and Right (Bilateral)	63 (18.2%)	53 (14.6%)
Palate	88 (26.7%)	158 (43.4%)
Other	3 (0.9%)	11 (3%)
Total	330 (100%)	364 (100%)
**Cleft position**		
Lip	47 (14.2%)	50 (13.7%)
Alveolar	1 (0.3%)	2 (0.5%)
Palate	88 (26.7%)	158 (43.4%)
Cleft lip and palate	174 (52.7%)	129 (35.4%)
Lip and alveolar	17 (5.2%)	14 (3.8%)
Other	3 (0.9%)	11 (3%)
Total	330 (100%)	364 (100%)

P-value < 0.001***

## Discussion

The prevalence rate for CLP in individuals utilising the specialised academic treatment centres in South Africa’s public sector was estimated to be 0.3 per 1000 live births and 0.4 per 1000 when the denominator was adjusted. This prevalence rate could be underestimated, because it excludes stillbirths, abortions and those children who might have died within the first three months of birth, or before seeking care. Nonetheless, this rate is also comparable to those found in Nigeria of 0.5 per 1000 live births [39] and higher than that of Ethiopia at 0.2 per 1000 live births [38]. In contrast, the prevalence rate reported in our study is lower than the rates reported for a population base birth defects registries from 30 countries from 54 international craniofacial registries during the period 2000 to 2005, where the overall prevalence of CLP was 1.0 per 1000 [51].

We also estimated the prevalence of orofacial clefts in each province. The prevalence rate ranged from a low of 0.1 per 1000 in the Eastern and Northern Cape Provinces to a high of 1.2 per 1000 in Free State Province. The geographic location of the different provinces within South Africa, and their different cultural and ethnic factors could have contributed to the variability of orofacial cleft prevalence found in this study. Other studies have also found that geographic factors are associated with orofacial cleft predisposition, occurrence and treatment methods [52]. A study performed in Colorado, USA, showed that country of residence and place of birth, whether metropolitan or non-metropolitan, had greater range of difference for OFC occurrence [9]. Nonetheless, our study did not determine the reasons for the geographic difference in the prevalence rates. This area requires future research.

In many LMICs including South Africa, infectious diseases dominate the causes of infant and child mortality, and congenital anomalies account for a relatively small proportion of under-five mortality [53]. Nonetheless, children born with CLP require treatment in the public sector that will start at infancy, and extend into late adolescence, or early adulthood. The affected individuals, their families, and the public health care system feel the burden of care as reported by Bamford et al [54] that the proportion of under-five mortality due to congenital anomalies has increased. Our study findings could inform the proposed national surveillance system on congenital anomalies.

Clefts of the lip have been reported to be predominant in blacks [55]. A retrospective study in Tanzania reported individuals with higher proportions of cleft lip only [56] and similar reports from a study in Kenya [57] and Zimbabwe [58]. Our study also found a predominance of black Africans with orofacial clefts (OFC). However, it could be a reflection of the South Africa’s population demographic profile where the majority of the population are black Africans who utilise the public health sector [59], rather than ethnic differences, as found in studies in other countries [7]. Further research is needed to determine whether ethnic differences play a role in South Africa.

Orofacial clefts have been reported to be more predominant in males compared to females [39]. Our study found a female predominance, with the majority of females presenting with CP. This proportion of cleft type from our study was similar to that found in a Nigerian study, which reported more females with CP than males [39]. Our study findings could be because parents perceive CP as the mildest form of cleft since it is not visible on the outside and hence the parent does not delay seeking care [60]. However, general societal neglect for cleft palate can lead to decreased access to palatal surgery [56, 61], and furthermore, CP has implications for feeding, speech and jaw development and is usually associated with syndromes. This type of cleft is more likely to increase morbidity and mortality from poor feeding leading to child malnutrition and vulnerability to infectious diseases [62, 63].

Treatment and management of individuals with orofacial clefts vary depending on the type and severity of the cleft, the presence of associated syndromes, other birth defects and the child’s age. Cleft lip repair is proposed to be done at three months after birth [64]. The median age at consultation for our study was about three months with a relatively high interquartile range of 3 weeks to 13 months. A retrospective study of the epidemiology, clinical aspect and management of clefts in Burkina Faso reported that more than 60% of children presented for consultation when they were older than one year [65]. However, delays in first consultation will delay treatment and the individual may suffer physical impairment and societal relationships with potential long-term psychological effects, including behavioural problems and lack of social integration [66–68].

Our study used clinical records to review previously recorded data to update the prevalence of clefting in the public sector hospitals in South Africa. Clinical records have advantages as they enable a relatively easy and less resource intensive research approach to answering specific clinical questions. However, they have certain disadvantages including variation in the manner in which data has been gathered and recorded in thus limiting the extraction and interpretation of the variables, as well as records may be incomplete or lost in the course of time, leading to missing data [69]. Furthermore, the estimation of prevalence from hospital records of CLP exclude all stillbirths and miscarriages that would be possible to obtain from an active birth defect surveillance system, thus leading to a possible underestimation of the prevalence rate. Although some of these patients might have been referred from the private sector to the academic centres, none of the records indicated this referral. Nevertheless, we analysed CLP records to compute an estimated prevalence of OFC in South Africa’s public health sector and to provide a detailed description of cleft types from the specialised academic centres. Therefore, our study has provided updated information on the epidemiology of CLP in South Africa’s public sector, especially since the end of apartheid in 1994. The study findings provides baseline data that should inform the implementation of the planned active birth surveillance system.

## Conclusions

The study has generated new knowledge on the epidemiology and clinical profile of individuals with CLP in the South African public health sector. It is imperative for South Africa to establish an active birth surveillance system on congenital anomalies to enable comprehensive management of CLP individuals and to inform health service planning and policy.

## Supporting information

S1 File (DOCX)

S2 File (XLSX)
