# Supplementary material for: Epidemiology and clinical profile of individuals with cleft lip and palate utilising specialised academic treatment centres in South Africa
Source: PLoS One. 2019 May 9;14(5):e0215931. doi: 10.1371/journal.pone.0215931 (PMC6508722; doi:10.1371/journal.pone.0215931)
Supplement: S1 File — (DOCX) [file pone.0215931.s001.docx]

**Epidemiology and clinical profile of individuals with cleft lip and palate utilising specialised academic treatment centres in South Africa**

**S 1 File_ RECORD REVIEW FORM**

**For official use only**

| 1. 3 | Participant number |  | \|  \|  \|  \|  \| \| --- \| --- \| --- \| --- \| |
| --- | --- | --- | --- | --- | --- | --- | --- |
|  | Study site   - SITE 1 - SITE 2 - SITE 3 - SITE 4 - SITE 5 - SITE 6 - SITE 7 - SITE 8 - SITE 9 - SITE 10 - SITE 11 |  | \|  \|  \|  \|  \|  \|  \| \| --- \| --- \| --- \| --- \| --- \| --- \| |
|  | Date of review: | DD/MM/YY | \|  \|  \|  \|  \|  \|  \| \| --- \| --- \| --- \| --- \| --- \| --- \| |
| 1. Da | Date of birth | DD/MM/YY | \|  \|  \|  \|  \|  \|  \| \| --- \| --- \| --- \| --- \| --- \| --- \| |
|  | Date of discharge | DD/MM/YY | \|  \|  \|  \|  \|  \|  \| \| --- \| --- \| --- \| --- \| --- \| --- \| |
|  | Record complete |  | 🞎Yes…1  🞎 No…0  🞎 Other …9 |

**Section 1: background information**

| For official use only |  |  |  |
| --- | --- | --- | --- |
| \|  \| \| --- \| |  | Gender | 🞎 Male…0  🞎 Female…1 |
| \|  \| \| --- \| |  | Age at time of first  consultation (indicate 0 if at birth) | ________________ |
| \|  \| \| --- \| |  | Population group | 🞎 Black…1  🞎 Coloured…2  🞎 Indian…3  🞎 White…4  🞎 Other…9 (specify)…………………………. |
| \|  \| \| --- \| |  | Foreign National | 🞎 No…0  🞎 Yes…1 |
| \|  \| \| --- \| |  | Province of residence at birth | 🞎 EC…1  🞎 Free State…2  🞎 Gauteng…3  🞎 KZN…4  🞎 Limpopo…5  🞎 Mpumalanga…..6  🞎 North West…..7  🞎 Northern Cape …..8  🞎 Western Cape…..9  🞎 Other ………..…10 |

**SECTION 2: clinical information**

| For official use only |  |  |  |
| --- | --- | --- | --- |
| \|  \| \| --- \| |  | Type of defect | 🞎 Cleft lip…1  🞎 Cleft palate…2  🞎 Cleft lip & palate…3  🞎 Other…9, please specify………….. |
| \|  \| \| --- \| |  | Description of cleft | 🞎 Unilateral…1  🞎 Bilateral…2  🞎 Palate…3  🞎 Midline…4  🞎 Other…9, please specify …………….. |
| \|  \| \| --- \| |  | Cleft laterality | 🞎 Left…1  🞎 Right…2  🞎 Left & Right…3  🞎 Palate…4  🞎 Midline…5  🞎 Other…9, please specify …………….. |
| \|  \| \| --- \| |  | Position of the cleft | 🞎 Lip…1  🞎 Alveolar…2  🞎 Palate…3  🞎 CLP …4  🞎 Lip & alveolar…5  🞎 Other…9, (please specify)………………. |

**SECTION 3: treatment/ care**

| For official use only |  |  |  |
| --- | --- | --- | --- |
| \|  \| \| --- \| |  | Information on treatment on record | 🞎 No…0  🞎 Yes…1  🞎 Incomplete…2 |
| \|  \| \| --- \| |  | Number of consultations in previous 12  months | ________________ |
| \|  \| \| --- \| |  | Internal referrals described | 🞎 No…0  🞎 Yes…1  🞎 Incomplete…2 |
|  |  | Stage of treatment | 🞎 Plastic surgery…1  🞎 Maxillo-facial surgery…2  🞎 Orthodontics…3  🞎 Speech therapy…4  🞎 Psychology…5  🞎 Genetics…6  🞎 General Dentistry…7  🞎 ENT…8  🞎 Other…9 |

**end of form**
